# Clinical Cardiovascular Applications of Hyperpolarized Magnetic Resonance

**DOI:** 10.1007/s10557-020-06942-w

**Published:** 2020-02-05

**Authors:** Andrew J. M. Lewis, Damian J. Tyler, Oliver Rider

**Affiliations:** Department of Medicine, University of Oxford Centre for Clinical Magnetic Resonance Research (OCMR), Radcliffe, Oxford, UK

**Keywords:** Hyperpolarization, Magnetic resonance, Cardiovascular

## Abstract

Current cardiovascular magnetic resonance imaging techniques provide an exquisite assessment of the structure and function of the heart and great vessels, but their ability to assess the molecular processes that underpin changes in cardiac function in health and disease is limited by inherent insensitivity. Hyperpolarized magnetic resonance is a new technology which overcomes this limitation, generating molecular contrast agents with an improvement in magnetic resonance signal of up to five orders of magnitude. One key molecule, hyperpolarized [1-^13^C]pyruvate, shows particular promise for the assessment of cardiac energy metabolism and other fundamental biological processes in cardiovascular disease. This molecule has numerous potential applications of clinical relevance and has now been translated to human use in early clinical studies. This review outlines the principles of hyperpolarized magnetic resonance and key potential cardiovascular applications for this new technology. Finally, we provide an overview of the pipeline for forthcoming hyperpolarized agents and their potential applications in cardiovascular disease.

## Background: the Need for Hyperpolarized Magnetic Resonance

**Fig. 1:**
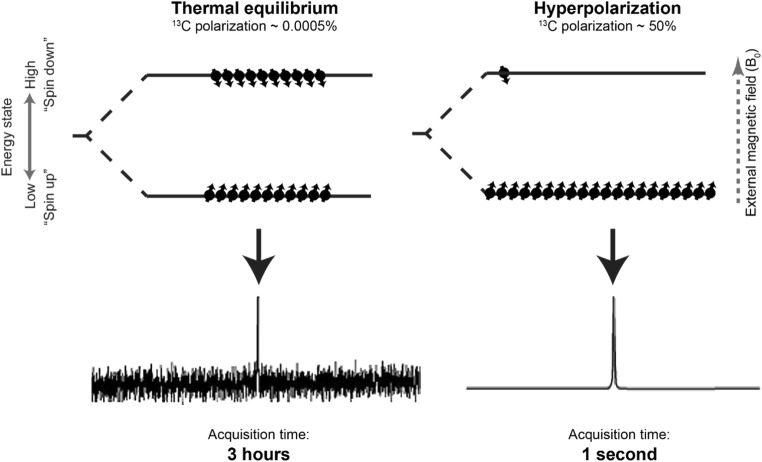
Comparison of ^13^C sample polarization arising from clinical field strength magnet at thermal equilibrium with subsequent hyperpolarized sample. Dramatically improved sample polarization resulting from hyperpolarization causes substantial improvement in spectral quality and acquisition time

The fundamental basis for signal in all magnetic resonance (MR) techniques lies in the interaction between certain atomic nuclei within the sample of interest and an external magnetic field. Spin active nuclei such as protons (which are highly abundant in biological tissues) are ordinarily randomly orientated when at biological temperatures and outside of a strong magnetic field (Fig. 1). However, when a strong external magnetic field is applied, as is the case within the bore of a magnetic resonance imaging (MRI) scanner, these spins separate into one of two groups. These groups can be described as either ‘spin up’ or ‘spin down’ (i.e. with or against the direction of the applied magnetic field according to a classical description) or alternatively as a high or low energy state (according to a quantum mechanical description). All magnetic resonance techniques create signal by inducing bulk nuclear transitions between these two states by applying and withdrawing radiofrequency energy, and the resulting signals are processed in order to create an image or spectrum.

Crucially, at clinically relevant magnetic field strengths and biological temperatures, there is a very small difference in the sizes of the populations of nuclei occupying the high and low energy states, with the low energy state slightly favoured. The magnitude of the difference in the sizes of the populations (i.e. the excess of spins in the low energy state) is referred to as the sample polarization. This sample polarization is the key determinant of the signal available to a magnetic resonance experiment since other bulk spin transitions effectively null the signal arising from each other.

The degree of sample polarization is determined by parameters including the external magnetic field strength, the sample temperature and the gyromagnetic ratio of the nucleus. Polarization can be estimated quantitatively using the Boltzmann distribution equation where *k*_B_ is the Boltzmann constant, Ɣ is the nuclear specific gyromagnetic ratio, ħ is the Planck constant divided by 2π, *B*_0_ is the field strength of the magnet and *T* is the sample temperature:

$$ \frac{N_{\mathrm{low}\ \mathrm{energy}}-{N}_{\mathrm{high}\ \mathrm{energy}}\ }{N_{\mathrm{low}\ \mathrm{energy}}+{N}_{\mathrm{high}\ \mathrm{energy}}}=\tan h\left(\frac{\upgamma \mathrm{\hbar}{B}_0}{2{k}_BT}\right) $$

For a population of protons at biological temperature and a field strength of 1.5 T, the sample polarization estimated using the Boltzmann distribution is only 0.00099%. This means that only a tiny fraction of the available nuclei contribute signal to the magnetic resonance experiment, explaining why these techniques have an intrinsically limited signal-to-noise ratio and why the potential improvements in signal achieved by increasing the field strength of the magnet are limited. Furthermore, the expense of an MR system increases greater than linearly with the field strength of the magnet, and ultra-high field systems are associated with further intrinsic limitations due to a greater heating effect from radiofrequency energy deposition; 7 T is currently the highest field strength used for clinical examinations. For the purpose of imaging the cardiovascular system, this limitation is mitigated by the very high abundance of protons within biological tissue. However, when studying nuclei other than protons, low sample polarization at even the highest available clinical field strengths becomes the fundamental barrier to magnetic resonance assessment and represents an important opportunity to overcome the lack of signal.

## Hyperpolarization Using the Dynamic Nuclear Polarization Method

Hyperpolarization refers to a range of techniques which can overcome low sample polarization in order to create contrast agents with huge increases in magnetic resonance signal [1]. For cardiovascular purposes, the key hyperpolarization technique is called dynamic nuclear polarization (DNP) [2], though other forms are used when creating, for example, hyperpolarized gases for assessment of the respiratory system or for other purposes.

In order to create a hyperpolarized agent, DNP involves the mixing of a molecule of interest containing an MR active nucleus (usually the stable isotope ^13^C which, unlike the more abundant but non-MR active ^12^C, has half integer spin as the sum of the number of protons and neutrons is odd) with a source of free electrons called a radical. This sample is then cooled to low temperatures of around 1 K within a strong magnetic field (usually 3–5 T though higher field strength polarizers are available). These conditions result in near unity polarization of the free electron population, and by applying microwave energy at a specific frequency, this polarization can be ‘transferred’ to the ^13^C nucleus within the molecule of interest resulting in hyperpolarization of the sample. Using this method, the signal gains from hyperpolarized MRI can frequently be as high as 10,000-fold greater than can be achieved without hyperpolarization, representing a potentially transformative improvement and the ability to assess entirely new biological processes [3].

One important consideration for biological and clinical applications is the need to return the sample to a biological temperature prior to administration. When using DNP, this is achieved by melting the frozen sample very rapidly by injecting a heated solvent such as water. Since the hyperpolarization usually decays rapidly after melting (this occurs at an exponential rate that is dependent upon the T_1_ time of the molecule), the molecule is then administered as soon as possible afterwards. It is for this reason that experimental and clinical DNP equipment is usually located near to MRI scanners.

One unique strength of magnetic resonance over other techniques is that different molecules can be differentiated according to a property called chemical shift. The chemical shift of a nucleus is determined in part by the surrounding chemical structure and the degree of electron shielding of the nucleus. Importantly, this offers the potential for hyperpolarized MR to assess the biological interconversion of substances in vivo, as downstream metabolites of a hyperpolarized agent will generally have a different chemical shift. This represents a unique advantage of the technique over technologies such as positron emission tomography (PET) which generally assesses tracer uptake rather than metabolism [4]. When combined with the fact that hyperpolarization of most ^13^C-labelled biological molecules generally lasts for little more than seconds to minutes after melting, the application of hyperpolarized MR is well suited to the study of rapid enzymatic processes in vivo such as those seen in metabolism.

Currently, the most carefully studied and clinically promising molecule for metabolic and other cardiovascular applications with hyperpolarized MRI is ^13^C-labelled pyruvate, which will be the focus for the majority of this review, though a strong pipeline of molecules in preclinical development will also be translated to human use in the near future.

## Cardiac Energy Metabolism and Hyperpolarized [1-^13^C]Pyruvate

In order to be suitable for use as a hyperpolarized MR probe for metabolism, the molecule of interest must have a sufficiently long T_1_ time to retain a meaningful degree of hyperpolarization during dissolution, administration and metabolism and must form a glass-like substance when frozen in the hyperpolarizer. In order to be of biological interest, the molecule should ideally also be at an important point of metabolic control to provide information about a biological mechanism of interest. These complex and sometimes competing requirements have so far been most successfully combined with the hyperpolarization of pyruvate, which occupies a central position within mammalian metabolism [5].

**Fig. 2:**
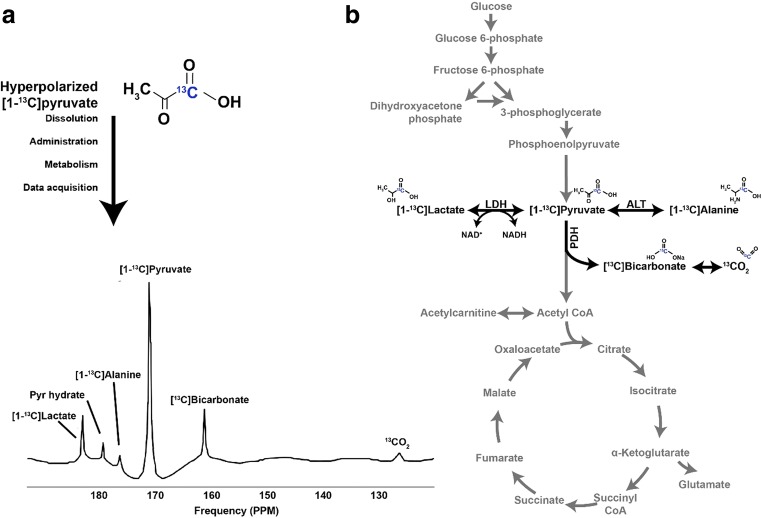
**a** Hyperpolarized [1-^13^C]pyruvate molecule and resulting spectrum following label incorporation in the heart using hyperpolarized [1-^13^C]pyruvate. **b** Biochemical basis for label exchange and resulting spectrum in the heart. LDH, lactate dehydrogenase; ALT, alanine aminotransferase

The pyruvate molecule is a three-carbon chain, and is the end product of glycolysis, linking glucose uptake to the tricarboxylic acid (TCA) cycle. Hyperpolarized [^13^C]pyruvate MR can be performed with a ^13^C label at either the first ([1-^13^C]) or second ([2-^13^C]) position of pyruvate (less commonly at both, [1, 2-^13^C]), and this enables measurement of flux through enzyme-catalysed pathways downstream (Fig. 2).

Pyruvate has a central role in cardiac energy metabolism. In order to fulfil its mechanical function as a pump, the heart generates and consumes chemical energy in the form of adenosine triphosphate (ATP). ATP is synthesised within mitochondria in a process driven by an electrochemical proton gradient produced by electron transfer in the form of the reducing equivalents, nicotinamide adenine dinucleotide (NADH) and flavin adenine dinucleotide (FADH). The sources of reducing equivalents for ATP synthesis are dietary fuels, including fatty acids and carbohydrates, and, to a lesser extent, amino acids and ketone bodies. In the normal heart in the fasted, resting state, the majority of ATP is derived from the beta oxidation of free fatty acids (overall 70–90% of ATP synthesis). Almost all the remainder is derived from the oxidation of pyruvate, with a small contribution from the process of glycolysis. In the fed state or during increased workload or hypoxia, there is a significant and adaptive increase in the relative contribution of carbohydrate metabolism to ATP generation.

There are three major metabolic fates of the [^13^C] label in hyperpolarized [1-^13^C]pyruvate which can be exchanged into [1-^13^C]alanine, [1-^13^C]lactate and [^13^C]bicarbonate pools in reactions catalysed by alanine aminotransferase, lactate dehydrogenase and pyruvate dehydrogenase, respectively (Fig. 2). ^13^CO_2_ equilibrates with [^13^C]bicarbonate in the pH-dependent carbonic anhydrase reaction. Of particular interest is pyruvate dehydrogenase because it is an enzyme which occupies a critical physiological role at a key point of metabolic control. Flux through pyruvate dehydrogenase (PDH) can be assessed by measuring the rate of ^13^C label incorporation from [1-13C]pyruvate into [^13^C]bicarbonate [6, 7]. Hyperpolarized magnetic resonance using [^13^C]pyruvate can arguably provide a more comprehensive overview of energy metabolism at the level of carbohydrate oxidation than is possible with any competing imaging technology.

## Pyruvate as a Molecule for Human Administration

In addition to its favourable properties for hyperpolarization and metabolic importance, pyruvate has a very good human safety profile. Pyruvate has been trialled with therapeutic intent in cardiovascular diseases at supraphysiological doses far in excess of those required for hyperpolarized MR studies with no significant safety issues demonstrated, including during direct and prolonged intracoronary infusion [8].

For the purposes of clinical administration of intravenous hyperpolarized [1-^13^C]pyruvate, a rapid quality control check to verify important safety parameters such as temperature and pH is performed prior to administration [9]. Although technically challenging, this approach has satisfied regulatory bodies internationally, and human studies using hyperpolarized pyruvate are now underway in several countries.

**Fig. 3:**
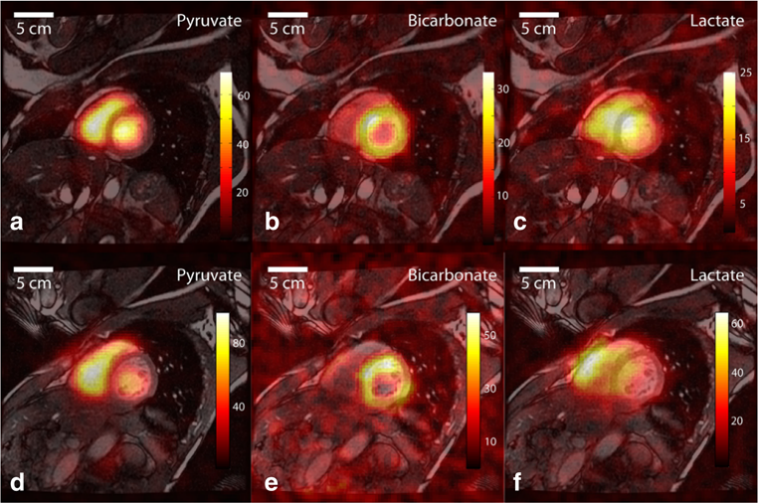
Reproduced without changes under Creative Commons licence from Cunningham et al [13]. Initial human experience using hyperpolarized [1-^13^C]pyruvate to image the heart. Pyruvate and its downstream metabolites can be detected with high spatial resolution. Representative ^13^C images displayed as colour overlays on top of greyscale anatomic images in a mid-left ventricle (LV) slice from two subjects (top row **a**–**c** and bottom row **d**–**f**, respectively). The [1-^13^C]pyruvate substrate was seen mainly in the blood pool within the cardiac chambers (**a** and **d**). Flux of pyruvate through the pyruvate dehydrogenase complex is reflected in the ^13^C-bicarbonate images (**b** and **e**), with signal predominantly in the wall of the LV. The [1-^13^C]lactate signal (**c** and **f**) appeared with a diffuse distribution covering the muscle and chambers

In preliminary “first-in-man” human studies, hyperpolarized pyruvate was shown to detect abnormal metabolism in prostate cancer tumours [10, 11]. Furthermore, hyperpolarized pyruvate has now been administered in order to evaluate the brain [12] and cardiovascular system [13] in healthy subjects, and the feasibility of detecting [1-^13^C]pyruvate and the downstream metabolites [1-^13^C]lactate and [^13^C]bicarbonate at high spatial resolution has been established (Fig. 3).

One advantage arising from the rapid magnetisation decay of hyperpolarized agents is that the imaging and acquisition are performed rapidly. From the perspective of the patient, a hyperpolarized study therefore adds as little as 5 min to the imaging study in addition to a change of MR coils. No major side effects or adverse events have ever been reported from clinical administration of hyperpolarized [1-^13^C]pyruvate, though some participants report a transient metallic taste during injection. The injection is performed via a power injector and intravenous extension line in a manner analogous to the administration of existing gadolinium-based contrast agents.

Having established feasibility, the next step is to define the clinical utility of hyperpolarized magnetic resonance to detect disease states and to identify the priority areas where it can add clinical value over existing imaging techniques [14]. The identification of the ‘killer application’ for clinical cardiovascular imaging will depend to a significant degree upon the results from early proof-of-concept studies, though there is good reason to believe that important applications will exist in ischaemic heart disease for the assessment of ischaemia, perfusion and viability. Novel applications as a probe to guide metabolic modulation therapy in failing hearts or to detect inflammation within the myocardium remain in experimental phases, though again appear to offer important potential.

## Hyperpolarized Pyruvate as a Probe for Cardiac Ischaemia

Ischaemic heart disease remains a leading cause of death worldwide [15]. The development of better techniques to diagnose and assess ischaemic heart disease and to better identify patients who might benefit from therapy is therefore an important and relevant goal. Many techniques are currently available to detect findings which might suggest myocardial ischaemia. These include coronary angiography, perfusion scintigraphy, perfusion MR, electrocardiography and wall motion assessments with and without stress among others. The value of such assessments is now in question following the presentation of the International Study of Comparative Health Effectiveness With Medical and Invasive Approaches (ISCHEMIA) trial, in which intervention to coronary stenoses associated with imaging or electrocardiographic evidence of ischaemia did not improve a composite clinical outcome measure at 5 years (currently unpublished). Although the potential reasons for this result are numerous and will be vigorously debated, it is clear that there is a need and opportunity to develop newer non-invasive tests which could more robustly define links between symptoms and specific coronary lesions, and guide more precise and effective intervention in stable coronary disease.

In rethinking our approach to cardiovascular ischaemia imaging, one hypothesis is that the ideal non-invasive test should provide a more fundamental measure of ischaemia than existing surrogate markers. For example, although imaging measures of myocardial perfusion will detect the earliest events in the ischaemic cascade, invasive or non-invasive measures of hypoperfusion do not necessarily correspond to symptomatic benefit following revascularisation and may not always be associated with true myocardial ischaemia [16]. Furthermore, a quantitative measure of earlier and more fundamental measures of ischaemia could be preferable in detecting late downstream consequences of ischaemia such as hypokinesia or electrocardiographic abnormalities. Hyperpolarized [1-^13^C]pyruvate may have a role in assessing the earliest molecular responses of the myocardium to ischaemia [17].

The heart is a transducer of chemical to mechanical energy and requires a continuous and matched supply of metabolic fuels including fat and glucose in addition to oxygen in order to sustain pump function. The myocardial metabolic programme changes rapidly when this balance between cardiac work and substrate/oxygen delivery occurs, as is the case during ischaemia [18]. One key change during ischaemia is the accumulation of the reduced forms of cofactors including NADH in mitochondrial and then cytosolic spaces. This shifts cardiac energy metabolism away from its usual oxidative programme and towards oxygen-sparing anaerobic glycolysis. This response promotes the conversion of cytosolic pyruvate to lactate in order to preserve the cardiac energetic state and sustain pump function.

During ischaemia, both the shift away from oxidative metabolism and shift towards glycolytic metabolism in ischaemic segments could, in principle, be assessed using hyperpolarized [1-^13^C]pyruvate. For example, hyperpolarized [1-^13^C]pyruvate imaging could be performed at rest and during increased cardiac workload to document lactate accumulation. In addition to mapping regional ischaemia-induced lactate accumulation, it is also likely that early myocardial responses to ischaemia will include a switch away from oxidative metabolism and a corresponding drop in [^13^C]bicarbonate signal. In keeping with this hypothesis, hyperpolarized pyruvate studies during global ischaemia in a perfused heart model demonstrated a marked drop in bicarbonate signal and a marked accumulation of lactate [19]. Furthermore, these findings now have been corroborated in large animal models [20].

In addition to the ability to detect lactate, hyperpolarized pyruvate also offers the potential for the mapping of ischaemic myocardial pH shifts [21]. Although this has, in principle, been possible in the past using ^31^P MRS, it has been challenging to measure cardiac intracellular pH in vivo, because 2,3-diphosphoglycerate (2,3-DPG) in the ventricular blood ordinarily overlies the myocardial inorganic phosphate (Pi) peak, though newer ultra-high field approaches may overcome this. The principle underlying pH assessment using hyperpolarized pyruvate is different and is based on the carbonic anhydrase-dependent interconversion of hyperpolarized bicarbonate and carbon dioxide according to the Henderson-Hasselbalch equation [21–23]. Whether measures of bicarbonate, lactate, pH or a combination provide the optimal strategy for studying cardiovascular ischaemia using hyperpolarization is unknown but will be tested in future human studies.

Hyperpolarized MR therefore shows promise as a method which could provide an earlier and more fundamental assessment of cardiac ischaemia than has previously been possible with any other technique. Although unlikely to replace conventional techniques such as angiography or perfusion scintigraphy in the short- to mid-term, there is particular promise for the assessment of borderline or equivocal cases and as a research tool to better understand fundamental mechanisms of ischaemia, particularly in grey areas such as microvascular dysfunction.

It is important to note that parallel advances in cardiac MRI offer ever-improving images of the proximal coronary arteries, alongside the routinely available high-quality assessments of wall motion and viability to complement hyperpolarized metabolite imaging. In future, cardiovascular magnetic resonance (CMR) using hyperpolarized MRI could provide a ‘one stop shop’ for the assessment of coronary anatomy, perfusion, myocardial ischaemia and viability.

## Hyperpolarized Pyruvate as a Probe for Cardiac Viability

The aim of cardiac viability assessment is to better target coronary revascularisation therapy. Whilst segments of viable hibernating ischaemic myocardium can recover function following revascularisation, those that are non-viable do not [24]. A key pathophysiological difference is that cardiomyocyte cell membrane integrity is preserved in hibernating viable myocardium along with glucose metabolism, but these features are lost in non-viable infarcted tissue. Furthermore, the hypocontractility seen in hibernating myocardium can be partially or completely restored by improving blood flow or by reducing oxygen demand.

Although improvements in left ventricle (LV) wall motion in revascularised hibernating segments are well established, whether viability-guided revascularisation improves outcomes versus revascularisation with no viability testing is unclear [25]. This again may therefore be a clinically important area in which further insight from metabolic imaging using hyperpolarized [1-^13^C]pyruvate could be beneficial. Given the potential to image myocardial perfusion, contractility and oxidative and glycolytic carbohydrate metabolism using hyperpolarized [1-13C]pyruvate near-simultaneously, it is likely that additional insights into the biology of hibernating myocardium could be obtained. This may provide a novel and more fundamental assessment of myocardial viability than is possible with current techniques [26].

## Myocardial Perfusion Imaging Using Hyperpolarized Tracers

The investigation of suspected myocardial ischaemia using stress perfusion imaging is a core function of clinical CMR laboratories. Stress perfusion CMR provides excellent prognostic information in ischaemic heart disease [27], particularly when combined with other measures of LVEF and late enhancement, and can be used to guide revascularisation procedures though this is currently a topic of debate.

Current CMR techniques for assessing myocardial perfusion involve the measurement of proton signal intensity changes during the first and second pass of a gadolinium-based contrast agent (GBCA) both at rest and during stress using either vasodilators or inotropes. T_1_ weighted images are acquired across the myocardium, and the change in the signal intensity during GBCA (which shortens T_1_) infusion was detected. Perfusion defects appear as a region of subendocardial signal hypointensity when compared with myocardial segments with normal perfusion. One limitation of GBCA-based perfusion imaging approaches is that they usually provide a subjective or semi-quantitative assessment of myocardial perfusion. This is because the change in signal intensity with GBCA correlates linearly with the concentration of agent across only a relatively narrow range of concentrations. This has historically limited the potential for absolute quantitation of perfusion, though new mathematical approaches have been described which may overcome this [28].

In contrast to GBCA techniques, the ^13^C signal intensity recorded during a perfusion study with a hyperpolarized ^13^C agent correlates essentially linearly with the concentration of the contrast agent across a wide range. Because essentially no ^13^C signal arises from natural abundance in the body, artefact from underlying structures is greatly reduced. This could also help to overcome difficulties in imaging ‘balanced’ ischaemia with other approaches. This concept has been established in preclinical models using adenosine to induce hyperaemia. The hyperpolarized agent [^13^C]urea has many favourable properties as a potential perfusion agent including the fact that it is an endogenous and intravascular molecule [29]. Importantly, the frequency of microwave irradiation required for polarization of [^13^C]urea is sufficiently similar to that of [1-^13^C]pyruvate that these molecules can be co-polarized and co-administered, providing a single infusion for assessment of cardiac perfusion and metabolism [30]. This approach could be particularly valuable for the assessment of cardiac perfusion/metabolism coupling during myocardial viability assessment, with advantages in acquisition time and ionising radiation dose when compared with existing PET techniques.

## Cardiac Energy Metabolism in Heart Failure

The role of changes in cardiac energy metabolism and energetics in the development of heart failure has long been of interest. Myocardial energy metabolism is known to become abnormal in heart failure, though no therapy has been approved to treat heart failure via a direct metabolic mechanism [31]. This may in part reflect the heterogeneity of clinical heart failure syndromes and the absence of techniques which could be used to assess cardiac energy metabolism in patients at serial timepoints and in response to therapy. Hyperpolarized magnetic resonance could therefore have an important role as a research tool in order to better understand mechanisms of energy starvation in failing hearts.

**Fig. 4:**
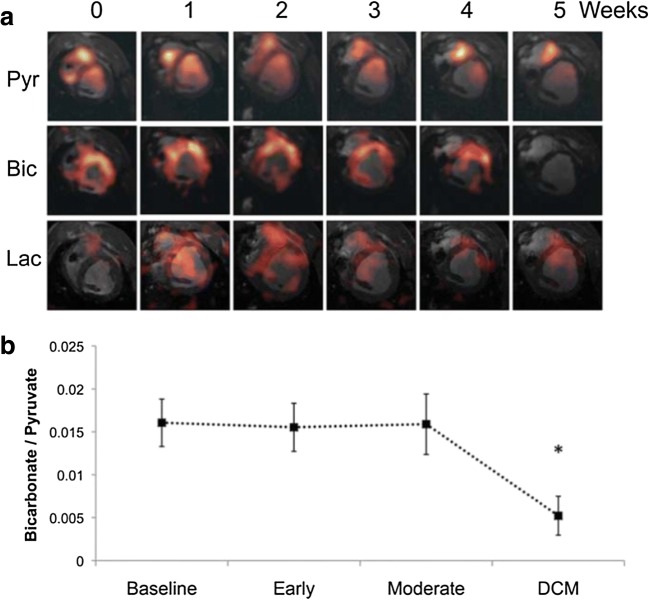
In a pig model of heart failure, hyperpolarized [1-^13^C]pyruvate magnetic resonance demonstrates impairment of PDH flux following the development of heart failure. Reproduced without changes under Creative Commons licence from Shroeder et al. [32]

In a pig model of heart failure induced by rapid pacing, hyperpolarized magnetic resonance using [1-^13^C]pyruvate and [2-^13^C]pyruvate revealed key metabolic changes during the early phases and transition to overt cardiomyopathy (Fig. 4) [32]. Early changes included impairment of Krebs cycle metabolism with a reduction in label incorporation into [13C]glutamate and reduction of cardiac energetic state assessed using ^31^P spectroscopy despite preservation of pyruvate oxidation. However, prolonged rapid pacing induced a transition to overt systolic dysfunction, further reduction in the glutamate/pyruvate ratio and impairment of PDH flux.

Corroborating these findings in a model of heart failure induced by myocardial infarction, impaired Krebs cycle activity was demonstrated using hyperpolarized [2-^13^C]pyruvate whilst PDH flux correlated with left ventricular ejection fraction [33].

Although preclinical models of heart failure with preserved ejection fraction are limited, associations between obesity and type II diabetes and diastolic dysfunction are well documented in humans with a possible link via abnormal carbohydrate metabolism. Using rodent models of diabetes, marked reductions in PDH flux have been demonstrated [7]. Furthermore, correction of this impairment of PDH flux pharmacologically using dicholoracetate (which activates PDH by inhibiting pyruvate dehydrogenase kinases) was associated with improvements in diastolic function [34]. These findings support a link between metabolic/energetic dysfunction and cardiac function [35] and provide a rationale for further investigation of the role of pyruvate dehydrogenase in heart failure associated with metabolic disease.

Although the widely used hyperpolarized pyruvate molecules provide an excellent assessment of cardiac carbohydrate metabolism, they do not directly assess fatty acid or ketone metabolism, both of which may also become dysregulated in heart failure. Hyperpolarized butyrate is a novel short chain fatty acid probe [36], whilst hyperpolarized [1-^13^C]acetoacetate and [1-^13^C]β-hydroxybutyrate can both probe ketone metabolism in the heart [37]. These molecules could be used in combination with pyruvate to provide a comprehensive and non-invasive assessment of cardiac energy metabolism.

A key rationale for developing a better understanding of metabolic changes in heart failure is the potential to understand whether pharmacological metabolic modulators could provide novel therapies to improve function. Numerous metabolic modulator drugs with activity at PDH or other enzymes have been proposed to treat heart failure by modulating metabolism. Fatty acid oxidation inhibitors including carnitine palmitoyl transferase 1 inhibitors are thought to promote a reciprocal increase in cardiac glucose utilisation [38] and have been proposed to be beneficial in small-scale human studies with heart failure or cardiomyopathy [39, 40]. Future human studies using hyperpolarized magnetic resonance could investigate metabolic perturbations in human heart failure of differing aetiologies and may identify the subgroups with the greatest potential to benefit from metabolic modulators. Furthermore, hyperpolarized magnetic resonance may have a role in measuring the myocardial metabolic responses to metabolic modulator therapy at an early phase of drug discovery.

Many metabolically active drugs, for example those used for the treatment of type II diabetes, have been found to have unexpected effects on cardiovascular risk and cardiac function. For example, although both glitazones and sodium glucose transport protein 2 inhibitors (SGLT2is) reduce serum glucose levels, glitazones are known to increase the risk of heart failure [41], whilst SGLT2is are protective against heart failure hospitalisations and mortality even the absence of diabetes [42]. A better understanding of how metabolically active drugs affect cardiac energy metabolism at an earlier phase of drug discovery could therefore be beneficial. Although these drugs have yet to be tested using hyperpolarized MRI, the effects of metformin upon cardiac energy metabolism have been investigated in rats. An unexpected effect to increase lactate signal was identified, likely reflecting a previously unrecognised redox effect [43], establishing the concept of using metabolic imaging to study metabolic effects of commonly used drugs upon the myocardium.

## Cardiac Injury and Immunology

**Fig. 5:**
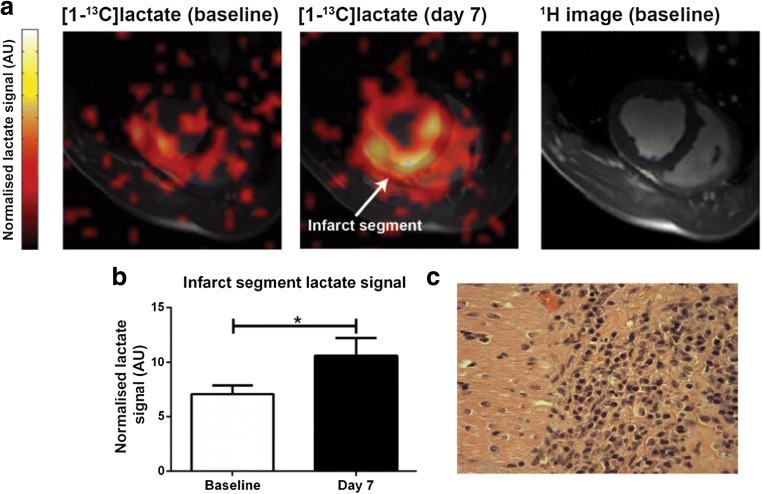
Imaging the local inflammatory response that follows myocardial infarction using hyperpolarized [1-^13^C]pyruvate. A lactate signature reflects monocyte/macrophage influx and metabolic reprogramming towards glycolysis. Reproduced without changes under Creative Commons licence from Lewis et al. [46]

The role of immune cells in the prevention, development and maintenance of cardiovascular diseases is increasingly recognised [44]. Current magnetic resonance techniques are able to resolve the consequences of dysregulated immune responses in inflammatory heart diseases leading to fibrosis or impairment of LV systolic function [45]. However, the assessment of disease phases characterised by active immune cell dysregulation is primarily through the indirect assessment of myocardial oedema using proton T_1_- and T_2_-based approaches. Hyperpolarization techniques offer several potential opportunities to more directly assess immune cell activity in the myocardium. First, using [1-^13^C]pyruvate, it is now possible to exploit the differences in metabolic phenotype between the myocardium (which is highly oxidative and hence produces energy via PDH) and immune cells (which are highly glycolytic when activated). As a result, the local inflammatory response which is known to follow myocardial infarction can be assessed via a resulting [1-^13^C]lactate signature (Fig. 5) [46]. The same effect has been established in other organ systems, including the brain [47, 48].

In addition to the potential to indirectly assess cardiac immune cell populations via their immunometabolic lactate signature, there is also the opportunity to directly probe myocardial necrosis using the novel tracer hyperpolarized [1, 4-^13^C_2_]fumarate [49]. This molecule provides a direct MR probe of active myocardial necrosis and as such could prove useful for assessing active phases of inflammatory diseases as well as a new tool to quantitate myocardial injury following other forms of injury. Hyperpolarized nanoparticles could also provide novel immunological disease-targeting agents [50].

## Angiography

Conventional angiographic techniques require invasive catheterisation and/or ionising radiation exposure. Although magnetic resonance angiography of coronary vessels continues to improve, it cannot yet match the performance of CT or invasive techniques. Hyperpolarized water is a promising hyperpolarized agent for angiographic applications [51]. In pilot work in animals, hyperpolarized water coronary angiography provided imaging with high temporal and spatial resolution [52]. Hyperpolarized water is therefore a promising strategy to improve the contrast-to-noise ratio of MRI of coronary arteries.

## Limitations of Hyperpolarized MRI and Comparison with Other Technologies

An important limitation of many hyperpolarized molecules with ^13^C nucleus is that their hyperpolarization tends to decay very rapidly following dissolution (usually seconds to minutes depending on the T_1_ of the molecule). This is a limitation for clinical applications where a quality control process is performed after dissolution and prior to administration to verify that key parameters including temperature and pH are within satisfactory ranges. Although this has been overcome with [1-^13^C]pyruvate, faster quality control processes will be required if other molecules such as ^13^C-labelled glucose, which have shorter T_1_ times, are to be clinically translated to human use [53]. Next, although ^13^C MR imaging sequences are in their infancy, it remains unclear whether hyperpolarized MR can achieve the same excellent spatial resolution as PET. Finally, clinical hyperpolarization facilities are only available in a limited number of centres worldwide, whereas many major hospitals are equipped with PET scanners with access to radioisotopes. Future clinical studies will define the situations in which the different modalities are preferable for answering the specific clinical or research question.

## Conclusions

Hyperpolarization results in a substantially increased MR signal which can overcome the sensitivity limitations of some existing multi-nuclear CMR applications. When applied to probes such as [1-^13^C]pyruvate, this has resulted in unparalleled real-time imaging of myocardial metabolic and other biological processes in vivo free from ionising radiation. With early human studies already underway, hyperpolarized MRI has clear potential to provide new insights over existing techniques in numerous cardiovascular diseases including ischemic heart disease, cardiac hypertrophy and heart failure. A strong pipeline of additional hyperpolarized agents has been developed, and many are suitable for clinical translation to human use in the near future.
